# Modeling the Intermediate Flow Regime in Flow‐Compensated Intravoxel Incoherent Motion MRI

**DOI:** 10.1002/mrm.70267

**Published:** 2026-01-26

**Authors:** Louise Rosenqvist, Mikael Montelius, Isabella M. Björkman‐Burtscher, Elina Petersson, Maria Ljungberg, Oscar Jalnefjord

**Affiliations:** ^1^ Department of Medical Radiation Sciences, Institute of Clinical Sciences, Sahlgrenska Academy University of Gothenburg Gothenburg Sweden; ^2^ Department of Radiology, Institute of Clinical Sciences, Sahlgrenska Academy University of Gothenburg Gothenburg Sweden; ^3^ Department of Radiology Sahlgrenska University Hospital Gothenburg, Region Västra Götaland Sweden; ^4^ Department of Medical Physics and Biomedical Engineering Sahlgrenska University Hospital Gothenburg, Region Västra Götaland Sweden

## Abstract

**Purpose:**

The intravoxel incoherent motion (IVIM) model is commonly used to separate the effects of motion related to diffusion and blood microcirculation (perfusion) on the MR signal. Depending on the encoding time (*T*), it is possible to probe the different temporal regimes of blood motion, which resemble a ballistic flow at short *T* and a pseudo‐diffusion at long *T*. The purpose of this work was to derive an encoding‐time‐dependent analytical model for flow‐compensated IVIM and to estimate the corresponding microvascular IVIM parameters in healthy brain.

**Theory and Methods:**

An encoding‐time‐dependent analytical IVIM model was derived for flow‐compensated/non‐flow‐compensated (FC/NC) double diffusion encoding (DDE) from the Langevin equation and validated using simulations. Eleven healthy participants were scanned to estimate microvascular IVIM parameters (blood velocity ν and blood correlation time τ) in healthy brain using the proposed model, with *T* = 50–100 ms.

**Results:**

The IVIM parameters were estimated to be *τ* = 123.1 ± 50 ms, *ν* = 1.51 ± 0.76 mm/s, perfusion fraction *f* = 4.75 ± 1.94%, and tissue diffusion coefficient *D =* 0.91 ± 0.32 μm^2^/ms in the healthy human brain, although simulations indicate a positive bias for *τ*. For very short/long *T*, the proposed model approaches established models for the ballistic/diffusive regimes. Pseudocode for the derivation of the analytical model is presented to facilitate a transfer to other gradient waveforms or pulse sequences.

**Conclusion:**

An encoding‐time‐dependent analytical IVIM model is presented for FC/NC DDE. In vivo results and simulations indicate that IVIM experiments with encoding times typical for clinical MRI scanners probe an intermediate to ballistic blood flow regime in the brain.

## 1 Introduction

Intravoxel incoherent motion (IVIM) imaging is a diffusion‐weighted imaging technique that utilizes low *b*‐values to estimate the signal contribution from incoherent blood flow (perfusion). A general model of the IVIM signal in the low to medium *b*‐value range can be written as [1]

(1) $$S/S_{0}=\left(1-f\right)\exp\left(-\mathrm{bD}\right)+fF_{p}\exp\left(-bD_{b}\right)$$

where $S/S_{0}$ is the diffusion‐weighted signal intensity normalized to the signal acquired at *b* = 0 s/mm^2^, f is the perfusion fraction, D is the extravascular diffusion coefficient, $D_{b}$ is the diffusion coefficient of blood and $F_{P}$ represents the signal attenuation from diffusion‐weighting due to capillary blood flow. The modeling of $F_{P}$ depends on the tissue's capillary architecture and the gradient waveform used for diffusion encoding (shape, strength, duration, etc.). The IVIM model, as originally presented by Le Bihan et al., considers the capillary network to consist of a series of randomly orientated straight capillary segments, for which two limiting cases for modeling the flow were defined [1]. In the first limit, the encoding time is short enough, or the flow is slow enough, for the flow through each capillary not to change direction during the encoding time, and signal attenuation arises due to the varying orientation of capillaries within a given MRI voxel. In the other limit, and perhaps the more common assumption in IVIM imaging, the flow changes direction multiple times during the encoding, resembling large‐scale diffusion, often called pseudodiffusion. These limits are often referred to as the ballistic and the diffusive regimes, respectively, and the dynamic transition between the two limits is referred to as the intermediate regime [2].

Acquiring the IVIM signal using flow‐compensated (FC) and non‐flow‐compensated (NC) gradient waveforms of varying encoding time can assist in differentiating the regimes. The signal attenuation caused by the spatial incoherence of capillaries can be recovered using FC gradients [3]. In the case of a ballistic or intermediate flow regime, FC gradients should yield a complete or partial signal recovery, respectively, compared to NC gradients. In contrast, FC gradients cannot rephase the signal attenuation caused by a pseudodiffusive flow due to the pseudorandomness of the motion. Since a ballistic regime presupposes a sufficiently short encoding time, increasing the encoding time allows the flow to change direction and transition into an intermediate and eventually diffusive regime. The signal acquired within the ballistic regime directly reflects the flow velocity since the motion in this regime remains unperturbed by randomness or temporal correlations. Outside the ballistic regime, the signal dynamics become increasingly influenced by the effects of temporal correlations, reflecting the average time the flow remains in one direction (i.e., correlation time). Disentangling the correlation time and velocity of the flow requires sampling at multiple encoding times.

To reach an analytical expression for the intermediate flow regime, Kennan et al. described the flow dynamics with a velocity correlation time (*τ*) [2]. The flow regimes may then be categorized based on the relationship between the encoding time (*T*) and *τ*, specifically *T*/*τ*, which represents the average number of times the flow changes direction during the encoding time. By incorporating the blood flow velocity, correlation time, and encoding time, the model is expected to describe the flow across all regimes [2]. Kennan et al. derived an expression for conventional diffusion encoding; however, it does not directly apply to other gradient waveforms, such as FC gradients. Wetscherek et al. estimated the correlation time and velocity in the upper abdomen using a numerical approach [4]. Identifying that the acquired phase of a flowing particle scales with *T*/τ and the average blood flow velocity for a given normalized gradient waveform, they developed a simulation‐driven model based on normalized phase distributions. Wu et al. estimated the intermediate flow characteristics in the mouse brain by introducing a three‐compartment IVIM model combining the two limiting cases using a weighted sum approach [5].

IVIM perfusion estimates offer a non‐invasive alternative for contrast agent‐based perfusion techniques, but inadequate modeling compromises the interpretation of these estimates. A few studies have shown that the common assumption of a pseudodiffusive flow does not always hold [4, 5, 6], suggesting that appropriate modeling demands a deeper understanding of the underlying biological properties. Estimating the blood flow velocity and correlation time may also provide a new contrast mechanism to reveal changes in the microstructure related to angiogenesis or treatment response.

In this study we present an analytical model for the IVIM signal in the intermediate flow regime applicable to a double diffusion encoding (DDE) pulse sequence to enable flow‐compensated gradients [7]. The model is evaluated through simulations and applied to in vivo data acquired at multiple diffusion encoding times (50–100 ms) to produce estimates of blood flow velocity and correlation time in the human brain.

## 2 Theory

### 2.1 Velocity Autocorrelation Function

**FIGURE 1:**
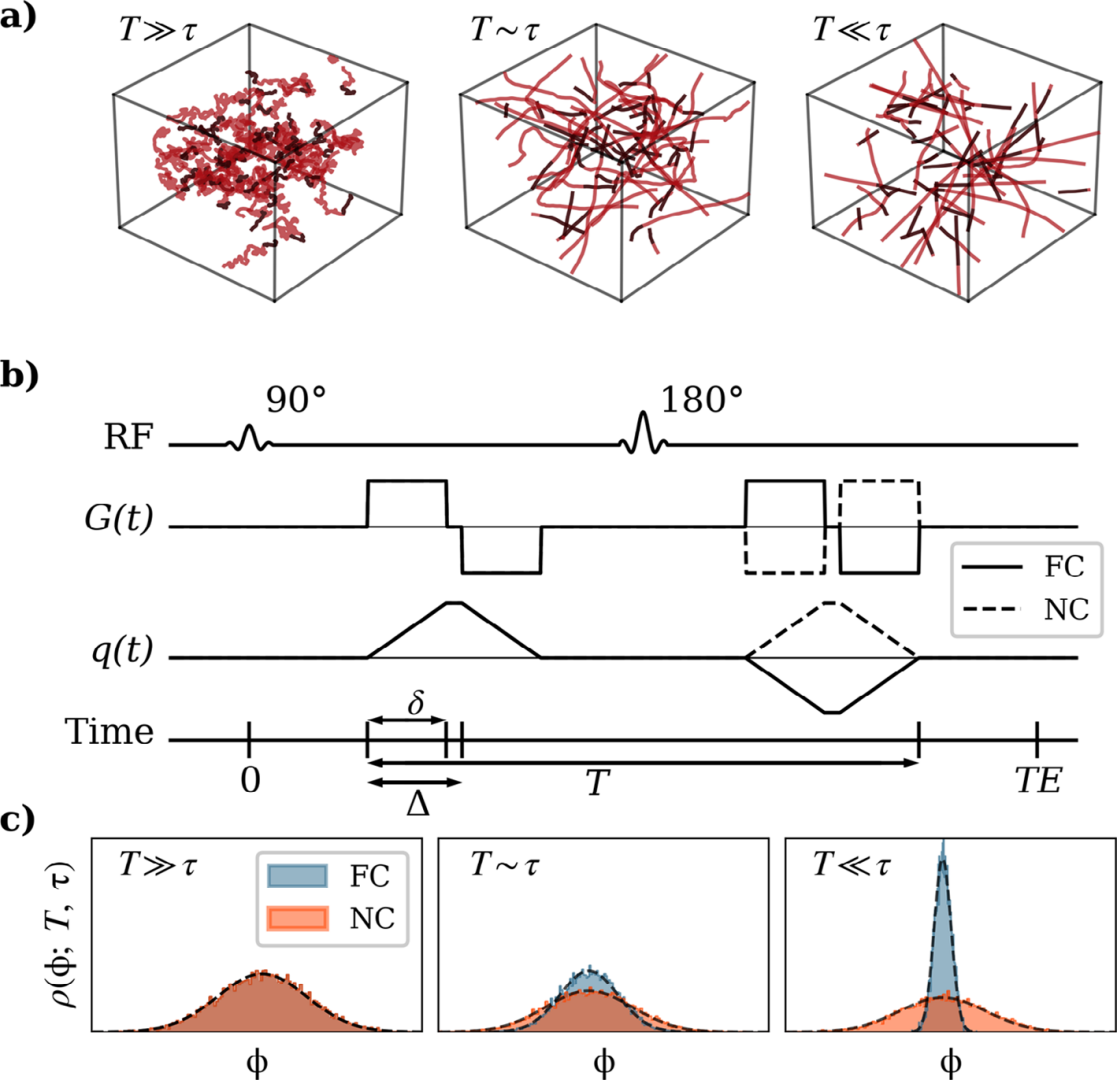
Simulation of intravoxel capillary flow of different tortuosity and the accumulated phase distributions under the influence of a flow‐/non‐flow‐compensated (FC/NC) double diffusion encoding (DDE) pulse sequence. (a) Random walks simulated using Equation (8) inside a 1 mm isotropic voxel, with varying correlation time *τ* such that *T*/*τ* = 10, 1, and 0, respectively. The three cases represent the diffusive (*T* **≫** τ), intermediate (*T*∼τ), and ballistic flow regimes (*T* **≪** τ). The darker part of the paths represents an example of the observed flow during *T* = 100 ms with *σ*_v_ = 2 mm/s. (b) Schematic illustration of the FC/NC DDE pulse sequence used in this study, showing RF‐pulses, gradient waveforms *G*(*t*), and dephasing factor *q*(*t*). The encoding time is defined as the duration from the beginning of the first bipolar gradient until the end of the second bipolar gradient. Under the influence of such gradients in one direction, the intravoxel flow simulated in (a) accumulates phase distribution ρ(ϕ;T,τ) as illustrated in (c). Black dashed lines in (c) represent a Gaussian distribution with zero mean and variance $\left\langle \phi^{2}\right\rangle$.

To model the capillary blood flow, we suggest starting from the Langevin equation

(2) $$d\vec{\nu }=-\frac{\vec{\nu }}{\tau }dt+\sqrt{\frac{2\sigma_{\nu }^{2}}{\tau }}d\vec{\xi }$$

where the velocity $\vec{\nu }$ is predicted to change by a conditional increment over the time interval dt, and stochastic fluctuations are introduced by $\vec{\xi }$, a scalar Wiener process with autocorrelation $\left\langle d\vec{\xi }\left(t\right)\cdot d\vec{\xi }\left(t^{\prime }\right)\right\rangle =\delta \left(t-t^{\prime }\right)dt$ [8]. In other words, the increments $d\vec{\xi }$ are uncorrelated for $t\neq t^{\prime }$ and have variance dt. The velocities are Gaussian distributed with zero mean and variance $\sigma_{\nu }^{2}$. According to this model, the velocities are exponentially correlated on a characteristic timescale *τ*, also referred to as the correlation time,

(3) $$\left\langle \vec{\nu }\left(t\right)\cdot \vec{\nu }\left(t^{\prime }\right)\right\rangle =\left\langle {\bar{\nu }}^{2}\right\rangle \exp\left(-\frac{\left|t^{\prime }-t\right|}{\tau }\right)$$

where $\left\langle {\bar{\nu }}^{2}\right\rangle$ is the average mean squared velocity [2]. Figure 1a illustrates how capillary tortuosity influences the correlation time of the velocity. Specifically, the correlation time increases as the trajectories become more ballistic, whereas shorter correlation times correspond to more diffusive dynamics.

### 2.2 MR Signal Model

Under the influence of a gradient magnetic field $\vec{G}\left(t\right)$, a particle moving along the trajectory $\vec{r}\left(t\right)$ acquires phase according to

(4) $$\phi =\gamma \int_{0}^{\mathrm{TE}}\vec{r}\left(t\right)\cdot \vec{G}\left(t\right)\;dt$$

where γ is the gyromagnetic ratio, and TE is the echo time. Integrating Equation (4) by parts and fulfilling the rephasing condition of a diffusion experiment ($\int_{0}^{\mathrm{TE}}\vec{G}\left(t\right)\;dt=0$), the acquired phase can be expressed in terms of velocity fluctuations

(5) $$\phi =-\int_{0}^{\mathrm{TE}}\vec{\nu }\left(t\right)\cdot \int_{0}^{t}\gamma \vec{G}\left(t^{\prime }\right)\;dt^{\prime }dt=-\int_{0}^{\mathrm{TE}}\vec{q}\left(t\right)\cdot \vec{\nu }\left(t\right)\;dt$$

where $\vec{q}\left(t\right)=\int_{0}^{t}\gamma \vec{G}\left(t^{\prime }\right)\;dt^{\prime }$ is the dephasing vector. In the case of linear diffusion encoding $\left(\vec{q}\left(t\right)={\left(q\left(t\right),0,0\right)}^{T}\right)$ [9], Equation (5) simplifies to

(6) $$\phi =-\int_{0}^{\mathrm{TE}}q\left(t\right)\nu \left(t\right)\;dt.$$

Here, ν denotes the velocity in the direction of the dephasing vector. For an ensemble average (denoted by angle brackets), Equation (6) can be expanded to express the phase variance using a velocity autocorrelation function,

(7) $$\left\langle \phi^{2}\right\rangle =\int_{0}^{\mathrm{TE}}\int_{0}^{\mathrm{TE}}q\left(t\right)q\left(t^{\prime }\right)\left\langle \nu \left(t\right)\nu \left(t^{\prime }\right)\right\rangle \;dt\;dt^{\prime }$$

where $\left\langle \nu \left(t\right)\nu \left(t^{\prime }\right)\right\rangle =\frac{\left\langle \vec{\nu }\left(t\right)\cdot \vec{\nu }\left(t^{\prime }\right)\right\rangle }{d}$ is the scalar velocity autocorrelation function for isotropic flow, and *d* is the dimensionality of the flow. That is, if the velocity autocorrelation in Equation (3) is assumed, $\left\langle \nu \left(t\right)\cdot \nu \left(t^{\prime }\right)\right\rangle =\frac{\left\langle {\bar{\nu }}^{2}\right\rangle }{d}\exp\left(-\frac{\left|t^{\prime }-t\right|}{\tau }\right)$. Assuming a Gaussian phase distribution with zero mean and variance $\left\langle \phi^{2}\right\rangle$, the attenuation of the MRI signal due to blood flow can be approximated as

(8) $$F_{P}=\exp\left(-\frac{\left\langle \phi^{2}\right\rangle }{2}\right).$$

The exact expression for Equation (8) depends on the velocity autocorrelation function, which we here assume is given by Equation (3), and *q*(*t*), which is dictated by the pulse sequence. We suggest the use of a single‐refocused DDE pulse sequence with FC or NC gradients (Figure 1b), resulting in the following expression:

(9) $$F_{P}=\exp\left(-\gamma^{2}G^{2}\left\langle {\bar{\nu }}^{2}\right\rangle \frac{\tau }{d}\left[\tau^{3}\left(\Psi +\Omega \right)-4\delta \tau^{2}+2\delta^{2}\left(\Delta -\frac{\delta }{3}\right)\right]\right)$$

with

(10) $$\Psi =2\exp\left(-\frac{∆+\delta }{\tau }\right)\left[\exp\left(\frac{\delta }{\tau }\right)-1\right]\left[2\exp\left(\frac{∆}{\tau }\right)+\exp\left(\frac{\delta }{\tau }\right)-1\right]$$

and

(11) $$\Omega =m{\left[\exp\left(\frac{\Delta }{\tau }\right)-1\right]}^{2}{\left[\exp\left(\frac{\delta }{\tau }\right)-1\right]}^{2}\exp\left(-\frac{T}{\tau }\right)$$

where *m* = 1 for NC and *m* = −1 for FC, δ is the gradient duration, and Δ is the gradient separation. For comparison, Equations ((9), (10), (11)) (with Ω = 0) can be rearranged to obtain the same expression derived by Kennan et al. for a pulsed gradient spin echo (PGSE) pulse sequence, scaled by a factor 2 to account for the second bipolar gradient in the DDE sequence. Equation (11) accounts for the combined effect of the two bipolar gradients, incorporating the total diffusion encoding time *T* of the diffusion experiment (see definition in Figure 1b). The derivation of Equations ((9), (10), (11)) was done using a Python package for symbolic math operations (sympy 1.12) with the algorithm outlined in Appendix A. The provided algorithm is applicable to other gradient waveforms.

The observed signal attenuation due to intravoxel incoherent flow during the encoding time can be characterized by *T/*τ, as illustrated in Figure 1a. Under the influence of FC or NC DDE gradients (Figure 1b), respectively, the intravoxel incoherent flow accumulates a phase distribution ρ(ϕ;T,τ) (Figure 1c). In the case of pseudodiffusive flow (*T* ≫ *τ*), the application of FC gradients has no rephasing effect on the phase dispersion relative to what is seen for NC gradients. In the intermediate regime (*T*∼τ), a contrast is obtained between FC and NC experiments. For completely ballistic flow (*T* ≪ τ), the phase dispersion of FC experiments is minimal, and the distribution approaches a delta function.

### 2.3 Limiting Cases

In the temporal limits of encoding the capillary flow, Equations ((9), (10), (11)) can be shown to approach previously published models. For short *τ* (diffusive regime), the first‐order terms of τ dominate, and Equation (9) reduces to the pseudodiffusive flow model proposed by Le Bihan et al. [1]

(12) $$F_{P}\approx \exp\left(-2\gamma^{2}G^{2}\delta^{2}\left(\Delta -\frac{\delta }{3}\right)\left\langle {\bar{\nu }}^{2}\right\rangle \frac{\tau }{d}\right)=\exp\left(-bD^{*}\right)$$

where $b=2\gamma^{2}G^{2}\delta^{2}\left(\Delta -\frac{\delta }{3}\right)$ for the DDE pulse sequence and $D^{*}=\frac{\left\langle {\bar{\nu }}^{2}\right\rangle \tau }{d}$ is the pseudodiffusion coefficient. Note that the diffusion coefficient is given by the autocorrelation function as $D=\frac{1}{d}\int_{0}^{\infty }\left\langle \nu \left(t\right)\nu \left(0\right)\right\rangle \;\mathrm{dt}$, which, e.g., is equal to $\frac{\left\langle {\bar{\nu }}^{2}\right\rangle \tau }{d}$ for an exponential autocorrelation function and $\frac{\left\langle {\bar{\nu }}^{2}\right\rangle \tau }{2d}$ for a linear one [2].

For long τ (ballistic regime), the τ^4^ term dominates. Evaluating Equations ((9), (10), (11)) by series expansion of Ψ and Ω up to the fourth order in τ gives

(13) $$\begin{array}{cc} F_{P} & \approx \exp\left(-2\gamma^{2}G^{2}\delta^{2}{∆}^{2}\frac{\left\langle {\bar{\nu }}^{2}\right\rangle }{d}\right)=\exp\left(-c^{2}\frac{\left\langle {\bar{\nu }}^{2}\right\rangle }{2d}\right)\;\left(\text{for}\;\mathrm{NC},m=1\right)\\ & \approx 1\;\;\left(\text{for}\;\mathrm{FC},m=-1\right) \end{array}$$

In a different approach, one may consider the velocity autocorrelation function for a ballistic flow, which reduces to $\left\langle \nu \left(t\right)\nu \left(t^{\prime }\right)\right\rangle =\left\langle {\bar{\nu }}^{2}\right\rangle$. Equation (7) then yields $\left\langle \phi^{2}\right\rangle =\frac{c^{2}\left\langle {\bar{\nu }}^{2}\right\rangle }{d}$, where $c=-\int_{0}^{\mathrm{TE}}q\left(t\right)\;dt$ is the flow‐weighting factor (with c=0 for FC acquisition). This is consistent with the model derived by Ahlgren et al. for ballistic flow using an FC/NC DDE pulse sequence [6]. Both limits (Equations 12 and 13, respectively) are consistent with the model derived by Kennan et al. for a PGSE pulse sequence when accounting for the fact that bipolar rather than monopolar diffusion encoding gradients were used in the current work.

## 3 Methods

### 3.1 MRI Acquisition

All experiments were performed on a clinical whole‐body 3 T scanner (Philips MR7700, Philips Healthcare, Best, Netherlands) using a 32‐channel head coil. A single‐refocused FC/NC DDE pulse sequence (Figure 1b) with single‐shot EPI readout was implemented using software enabling free gradient waveforms [10]. The gradient waveforms allowed FC to be disabled/enabled by inverting the polarity of the second bipolar gradient [6]. Common settings for all diffusion experiments were: TR/TE = 3700/180 ms, *T* = 50, 60, 85, 100 ms, six diffusion encoding directions (*x*, *y*, *z*, −*x*, −*y*, −*z*), diffusion gradient duration *δ* = 8.6 ms, diffusion gradient separation Δ = 10 ms, voxel size = 2 × 2 × 4 mm^3^, 1 mm slice gap, 17 slices, BW_PE_ = 27.2 Hz/pixel (posterior–anterior direction), SENSE = 1.9 (anterior–posterior direction), halfscan factor = 0.79, fat suppression by SPIR and gradient reversal, EPI factor 63 and effective echo spacing = 0.28 ms. Double‐sampled EPI was used to reduce ghosting artifacts [11]. *b*‐values were acquired in the order: 0, 200, 5, 100, 10, 30, 20 s/mm^2^. Dynamic stabilization of the *B*_0_ field was disabled to prevent striping artifacts [12]. A *b* = 0 image was acquired with a reversed phase‐encoding direction to enable correction for geometric distortions. The acquisition time for each DDE scan (FC/NC and specific *T*) was 5 min 14 s.

#### 3.1.1 Phantom Study

In order to validate the implementation of flow‐compensated gradient waveforms, a phantom was constructed to generate a laminar flow profile through the axial imaging plane. The phantom consisted of an 18‐mm inner diameter plastic pipe connected via water hoses to a pump (Type GP200, Grant Instruments, Cambridge, England) for continuous water flow. The imaging plane was placed 1 m beyond the pipe entrance. Water stored at room temperature was used. A stationary water phantom was included in the field of view as a reference. Phantom data were acquired with and without FC gradients. Phase contrast images were acquired before the IVIM imaging, measuring an average velocity of 2 cm/s. This corresponds to a Reynolds number R_e_ = 340, indicating that a laminar flow (R_e_ < 2300) was established [13].

#### 3.1.2 In Vivo Study

Brain data from 11 healthy participants (age 26 ± 7 years, 6 male and 5 female) were acquired using the imaging protocol described above. In addition, structural T1‐weighted 3D images were acquired (T1W‐turbo field echo, TR/TE = 7.6/3.5 ms, flip angle = 8°, FOV = 250 mm^2^, 1 × 1 × 1 mm^3^ voxels, acquisition time = 3 min 5 s). The Swedish Ethical Review Authority approved the study (ref no 2020‐00029), and all participants gave written informed consent before examinations.

### 3.2 Data Preprocessing and Analysis

The preprocessing of the diffusion‐weighted in vivo images included corrections for distortions, motion, and signal drift. The susceptibility‐induced distortion fields were estimated using TOPUP [14, 15] (FSL version 6.0.5) based on six repeated *b* = 0 images and one *b* = 0 image with reversed phase encoding direction and were applied to all in vivo data using APPLYTOPUP. Motion and eddy current‐induced distortions were corrected for using affine registration (eddy_correct) to an average of the six *b* = 0 images prior to running APPLYTOPUP. The six corrected *b* = 0 images were then used to apply a signal drift correction [16]. Lastly, the geometric average DWI signal was calculated from all encoding directions.

Parameter maps were estimated using voxel‐wise Bayesian inference on the proposed model (Equations (9), (10), (11)), with a Markov Chain Monte Carlo method to estimate posterior parameter distributions. The method used a voxel‐wise uniform prior distribution for each model parameter: D∈[0,3] μm^2^/ms, f∈[0,1], v∈[0,5] mm/s, τ∈[1,1000] ms, $S_{0}\in \left[0,2\cdot S_{\max}\right]$, where $S_{\max}$ is the maximum measured signal. A spatial prior distribution was used to inform neighboring voxels. The method is available at https://github.com/oscarjalnefjord/ivim/tree/intermediate_regime, and for more details, see Gustafsson et al. [17] The diffusion coefficient of blood was fixed to *D*_*b*_ = 1.75 μm^2^/ms [6].

White matter (WM) and cortical gray matter (CGM) segmentation was performed using FSL's FAST [18], and deep gray matter (DGM) segmentation using FIRST [19]. The T1‐weighted image was used as a reference for linear image registration (FLIRT) [20, 21] to register the segmentations to the DWI space. Subsequently, the segmentations were manually eroded using ITK‐snap (version 3.8.0) [22] to minimize partial volume effects. The parameter values presented were estimated using these segmentations across three consecutive axial slices. The uncertainty of parameter estimates is presented as the 95% confidence interval of the median, obtained through 10 000 bootstrap iterations.

### 3.3 Simulations

To validate the derived model, simulations were performed utilizing a discrete version of the Langevin equation (Equation 2),

(14) $$\nu \left(t+\Delta t\right)=\nu \left(t\right)\left(1-\frac{\Delta t}{\tau }\right)+\sqrt{\frac{2\sigma_{\nu }^{2}}{\tau }}\Delta \xi .$$

Initial velocities were randomized from a Gaussian distribution with zero mean and standard deviation *σ*_*ν*_ = 2 mm/s. The increments Δξ were randomized from a Gaussian distribution with zero mean and variance Δ*t* = 0.1 ms. The simulations were run with *N*_walkers_ = 20 000 random walkers, of which approximately 50 trajectories are visualized in Figure 1a. The resulting signal was calculated as

(15) $$F_{P}=\frac{1}{N_{\text{walkers}}}\sum_{j}\exp\left(i\phi_{j}\right)$$

where the phase contribution $\phi_{j}$ was calculated according to Equation (6), and the dephasing factor *q*(*t*) was defined as rectangular gradient waveforms with duration Δ = 10 ms and separation *δ* = 8.6 ms (Figure 1b).

The robustness to noise was assessed using synthetic IVIM data generated from Equations (1) and ((9), (10), (11)), with acquisition parameters matching those of the in vivo MRI acquisition, and with τ reflecting a diffusive (10 ms), intermediate (130 ms), and ballistic (800 ms) regime. The typical in vivo SNR was calculated voxel‐wise as the ratio of the mean to the standard deviation of the six TOPUP‐corrected *b* = 0 images, taking into account the averaging across encoding directions. The resulting SNR in the brain was ≈30–70. Rician noise was added for a range of SNR levels (20–2000), and parameters were estimated using the Bayesian fitting described above. An additional simulation, provided as supporting material (Supporting Information Figure S1), was performed to assess parameter estimation under different signal conditions, specifically using realistic IVIM parameters for liver and pancreas [4].

All simulations, data preprocessing, and analysis were run in Python (version 3.10.13) with packages numpy (1.26.0), nibabel (5.1.0), sympy (1.12), scipy (1.11.3), matplotlib (3.8.3), and pandas (2.1.1).

## 4 Results

### 4.1 Phantom Data

**FIGURE 2:**
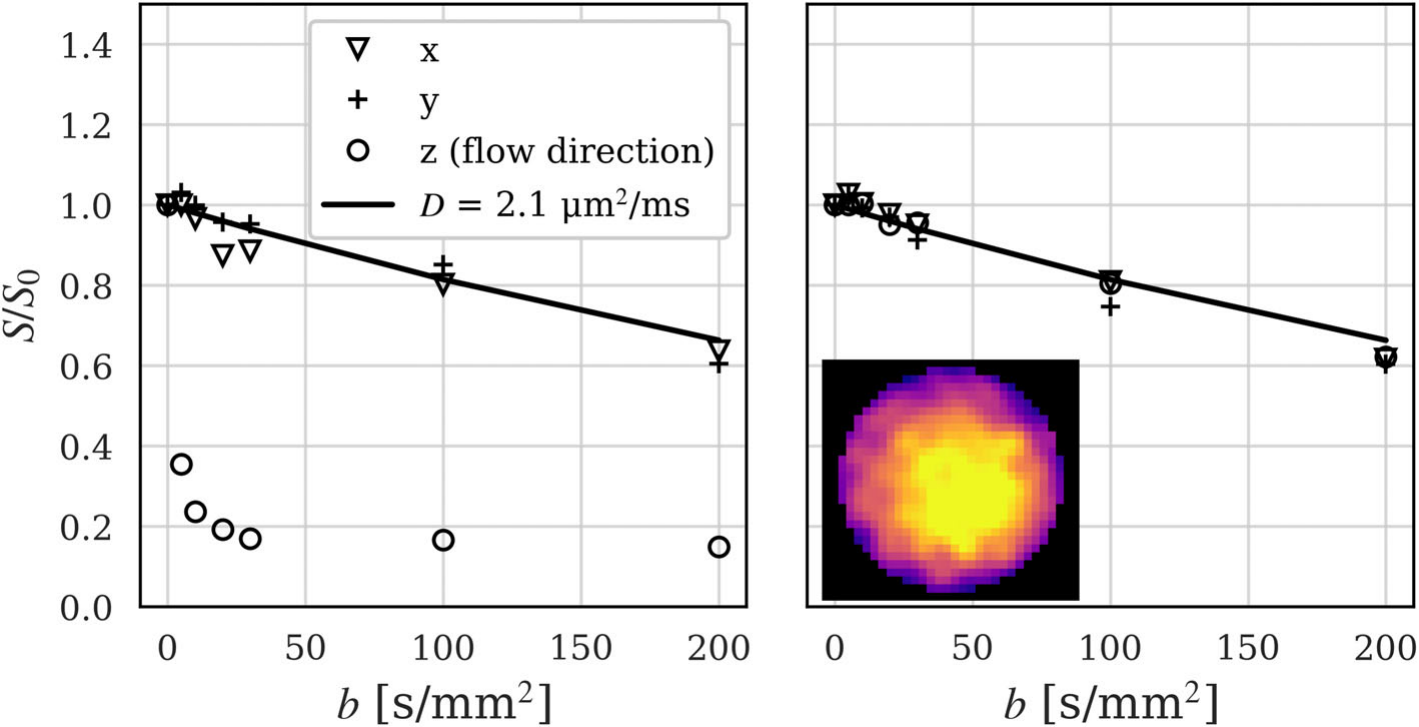
Gradient waveform validation experiments using a laminar water flow phantom. Diffusion‐weighted images were acquired using three orthogonal diffusion encoding directions (*x*, *y*, *z*) with flow‐compensation disabled (left) and enabled (right). As a reference, the estimated diffusion coefficient (*D*) of stationary water at room temperature is shown (solid line). A cross‐sectional phase contrast image of the flow phantom is presented to the lower left.

The validation of the FC gradient waveform revealed a contrast between FC and NC signals using diffusion gradients parallel to the flow direction (Figure 2), with a rapidly decaying signal using NC gradients and a recovered signal using FC gradients. A monoexponential signal decay was observed for diffusion encoding orthogonal to the flow direction, regardless of whether FC gradients were enabled or disabled.

### 4.2 Simulations

**FIGURE 3:**
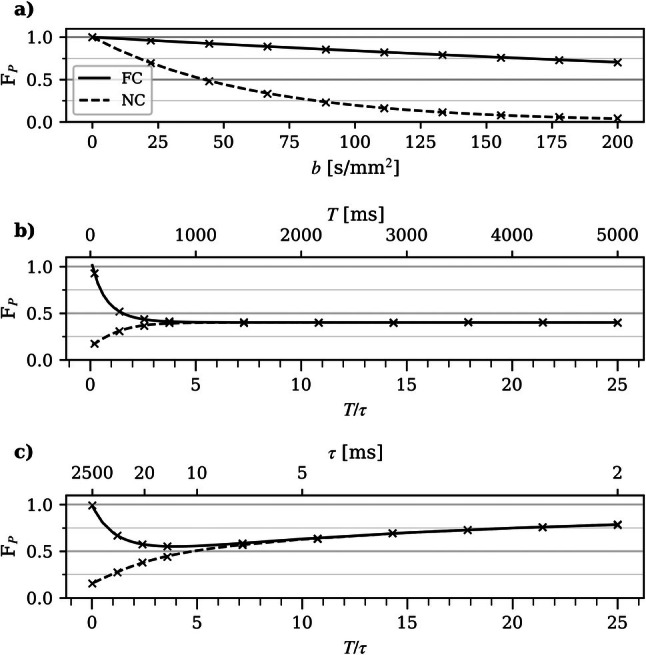
Analytical perfusion signal *F*_*P*_ using flow‐compensated (FC; solid) and non‐flow‐compensated (NC; dashed) gradients vs. (a) *b*‐value with *T*/*τ* = 0.4, (b) *T*/τ with *τ* = 200 ms and varying *T*, and (c) *T*/*τ* with *T* = 50 ms and varying τ. Markers represent simulated perfusion signal. Simulation values used were: *σ*_ν_ = 1.5 mm/s, time step Δ*t* = 0.1 ms, gradient duration *δ* = 8.6 ms, and gradient separation Δ = 10 ms.

Blood flow simulations, based on the Langevin equation with exponentially correlated velocities under the influence of an FC/NC DDE pulse sequence, yielded Gaussian phase distributions with increasing phase dispersion as *T*/τ increased (Figure 1). As illustrated in Figure 3, the simulated *F*_*P*_ signal and the analytical model (Equations (9), (10), (11)) agree well, both revealing contrasting FC and NC signals at low *b*‐values (Figure 3a) and for approximately *T*/*τ* < 3 (Figure 3b). The FC signal exhibits non‐monotonic behavior as a function of τ (Figure 3c). For short τ (*T*/*τ* ≥ 6), i.e., in the diffusive regime, the signal increases for decreasing τ as a result of the decreasing effective D*. On the other hand, for long τ (*T*/*τ* < 3), i.e., in the intermediate to the ballistic regime, the signal increases for increasing τ as a result of the increasing rephasing effect of the flow compensation.

**FIGURE 4:**
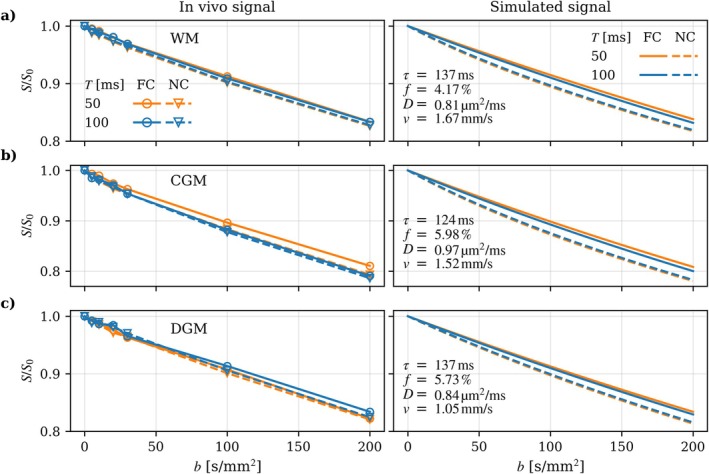
Signal attenuation curves for (a) white matter (WM), (b) cortical gray matter (CGM), and (c) deep gray matter (DGM) at *T* = 50 ms and *T* = 100 ms. The left column shows the in vivo signal for one subject, and the right column shows noise‐free simulated signal based on the corresponding in vivo estimates. To maintain figure readability, only two *T* curves are shown, and in vivo data (left) and fitted curves (right) are shown separately.

**FIGURE 5:**
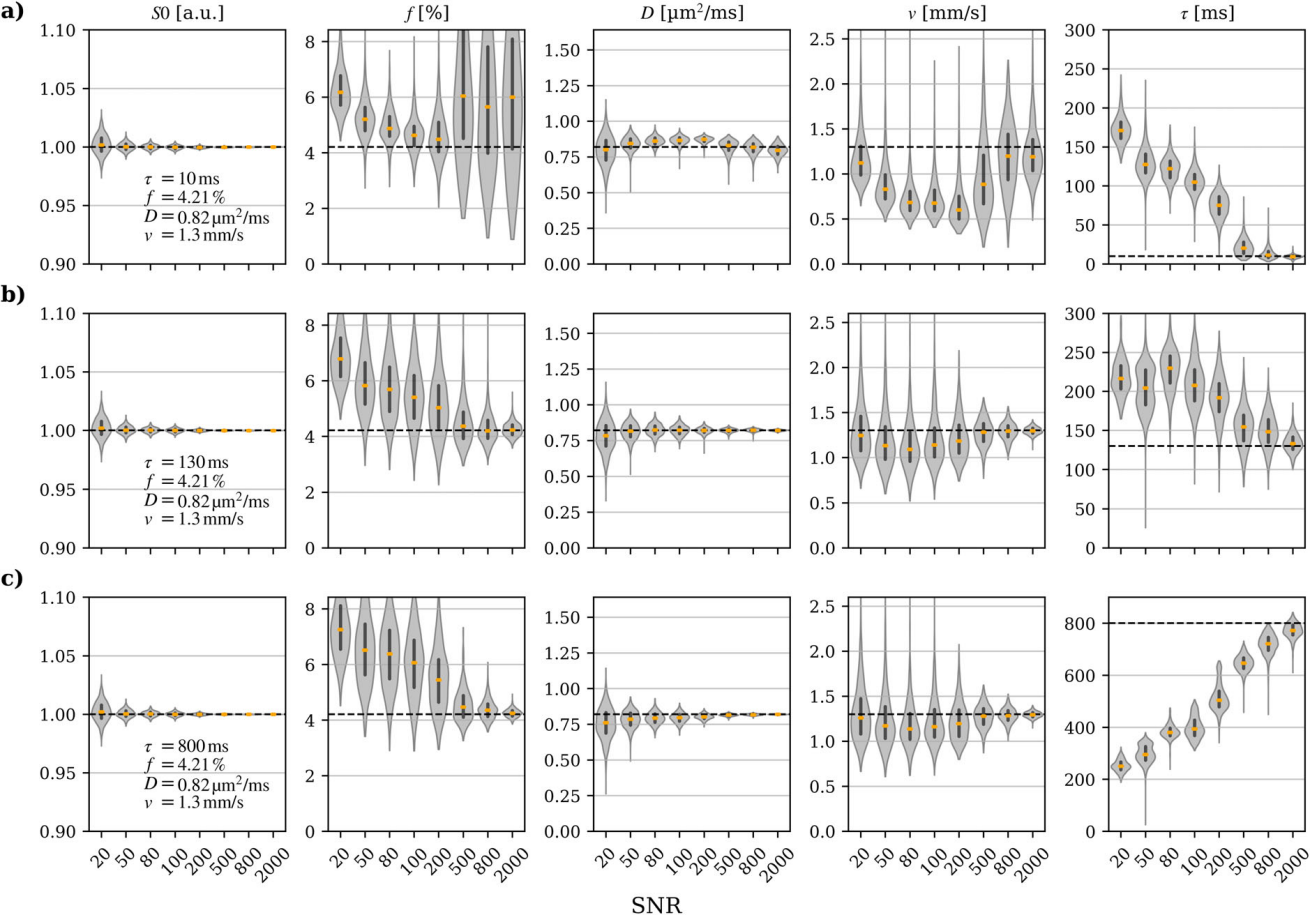
Estimated IVIM parameters for synthetic data at different SNR levels, with *τ* reflecting (a) diffusive regime, (b) intermediate regime, and (c) ballistic regime. The black dashed line shows the ground‐truth value, the black line shows the interquartile range, and the orange line shows the median. Additional simulations are provided using IVIM parameters realistic for the liver and pancreas [4] (Supporting Information Figure S1).

Figure 4 shows the in vivo IVIM signal vs. *b*‐value for WM, CGM, and DGM in one subject, and noise‐free simulated signal (Equations 1 and (9), (10), (11)) from the fitted parameters. A consistent separation between the FC and NC signals can be observed for both *T* = 50 ms and *T* = 100 ms, showing a rephasing effect of the FC gradients. CGM and DGM, which are more susceptible to partial volume effects, show greater variability. The IVIM parameter estimates derived from the simulated signal at different SNR levels and three different flow regimes are presented in Figure 5. The diffusion coefficient *D* showed robust estimates across flow regimes and SNR levels, with minimal bias and narrow variability. The perfusion fraction *f* displayed a consistent positive bias of approximately 1% in the intermediate regime, with the greatest spread observed in the ballistic regime at realistic SNR levels. The blood velocity *ν* was generally underestimated by 0.2–0.3 mm/s in the intermediate region, with the lowest bias in the ballistic regime. The correlation time τ showed low variability across all regimes, but a positive bias in the diffusive and intermediate regimes and a negative bias in the ballistic regime. At high SNR, parameter estimation converged towards the true values for all parameters in all regimes except for *f* and ν in the diffusive regime, likely caused by inherent overparameterization of the model in the diffusive regime. The simulations based on IVIM parameter values typical for the well‐perfused liver and pancreas (*f* = 30%) showed substantially less noise sensitivity with negligible bias and low variability even at relatively low SNR levels (Supporting Information Figure S1).

### 4.3 In Vivo Data

**FIGURE 6:**
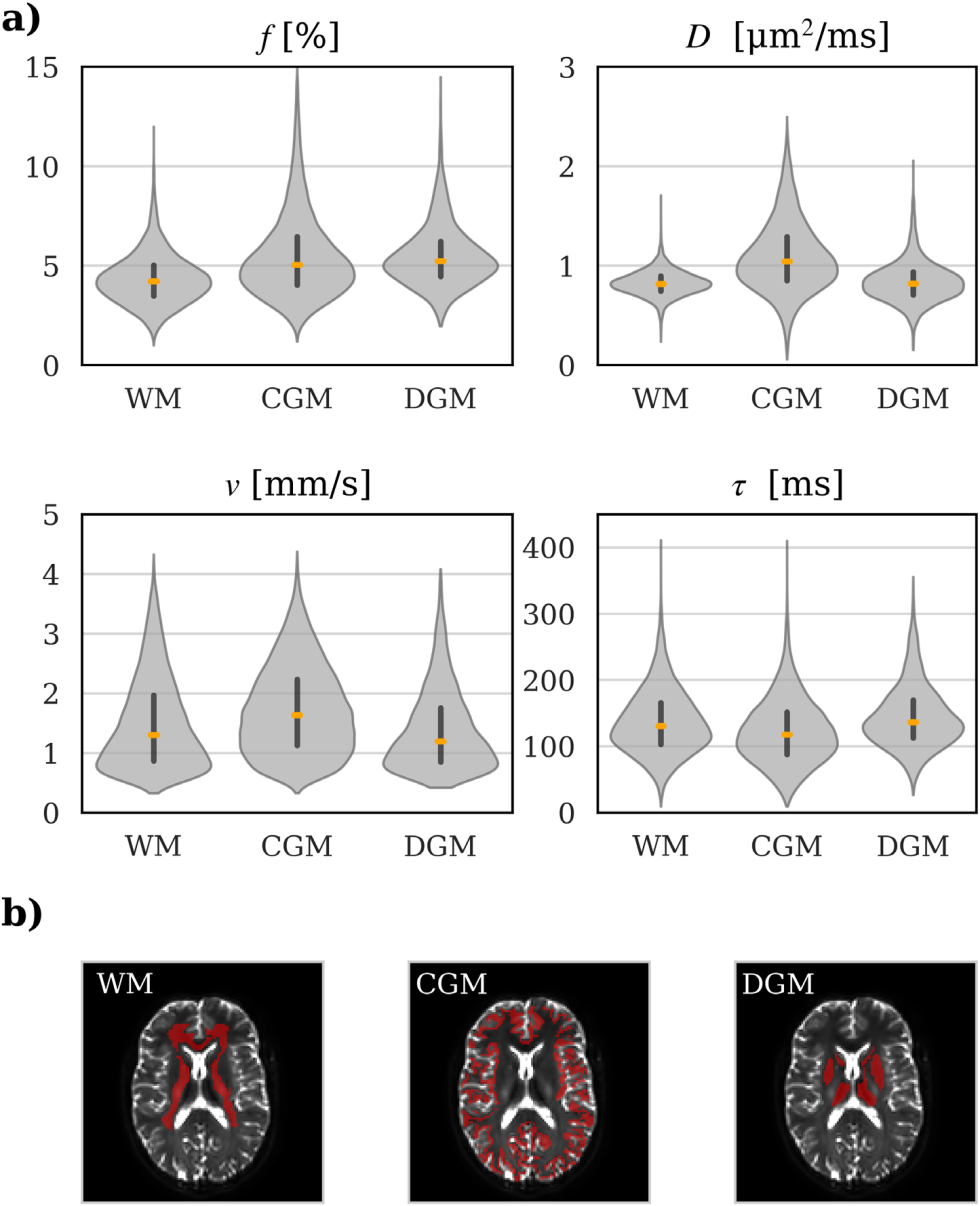
Violin plots of estimated IVIM parameters (*f*, perfusion fraction; *D*, diffusion coefficient; *ν*, blood flow velocity; *τ*, correlation time) in white matter (WM), cortical gray matter (CGM), and deep gray matter (DGM). (a) The violins reflect the distribution of samples, including the median value (orange line) and interquartile range (black line). (b) Example segmentation of WM, CGM, and DGM in one subject displayed on an axial *b* = 0 image.

**FIGURE 7:**
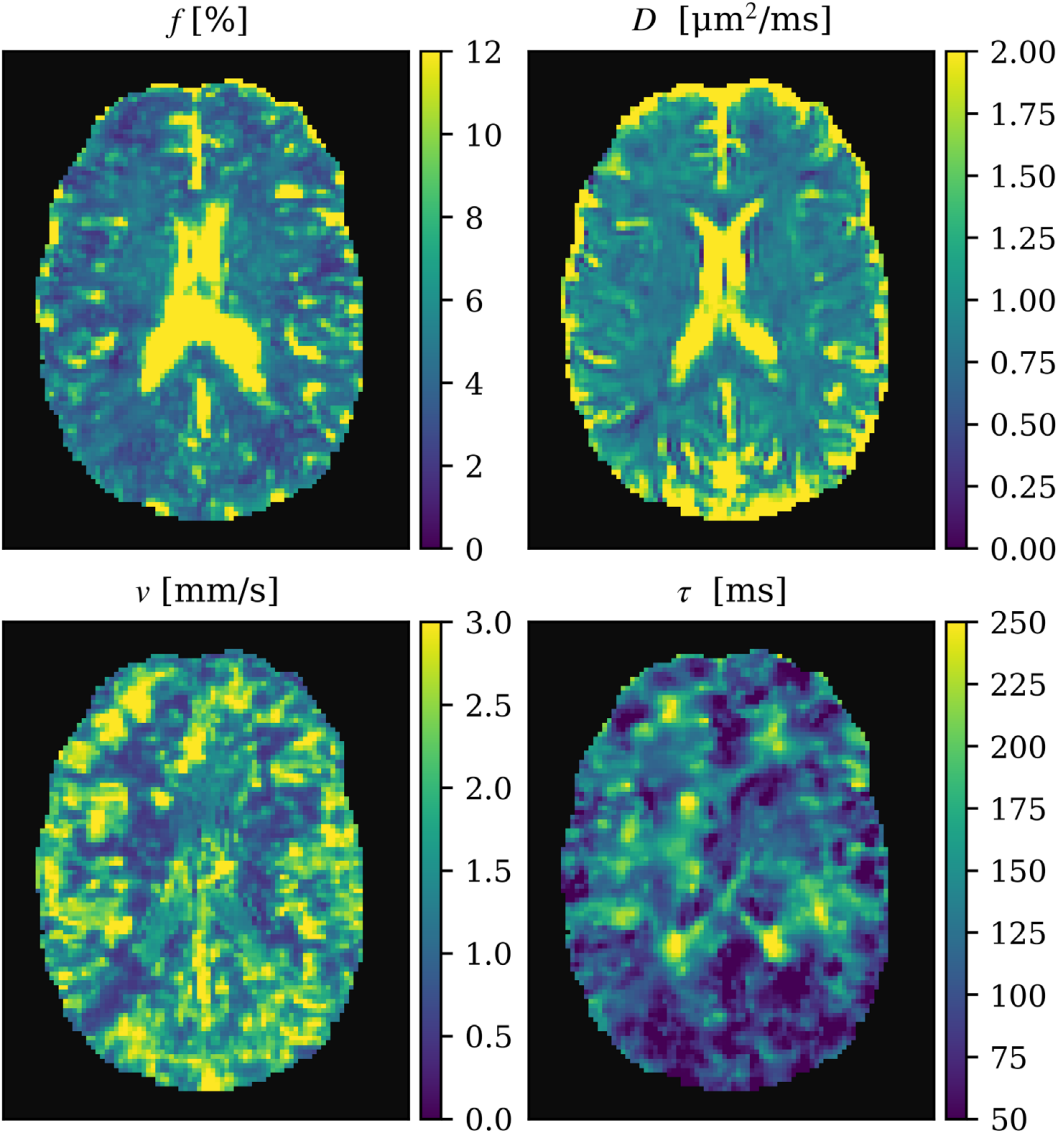
Example in vivo data of estimated IVIM parameter maps in an axial slice of the brain, showing *f*, perfusion fraction; *D*, diffusion coefficient; *ν*, blood flow velocity; *τ*, correlation time.

The median of IVIM parameters in the brain parenchyma was estimated to be τ = 123 ± 50 ms, *ν* = 1.51 ± 0.76 mm/s, *f =* 4.75 ± 1.94%, and *D* = 0.91 ± 0.32 μm^2^/ms. Specifically, τ and ν were estimated to 130 ± 49 ms and 1.30 ± 0.77 mm/s in WM, 117 ± 49 ms and 1.63 ± 0.75 mm/s in CGM, and 136 ± 47 ms and 1.20 ± 0.68 mm/s in DGM. Corresponding values for *f* and *D* are presented in Table 1. Figure 6 shows the distribution of estimated parameters across the 11 subjects for voxels segmented into WM, CGM, and DGM, and an example of the estimated parameter maps for one subject is shown in Figure 7.

**TABLE 1:** Median value ± standard deviation (95% confidence interval of the median) of estimated IVIM model parameters in segmented white matter (WM), cortical gray matter (CGM), and deep gray matter (DGM) across 11 subjects.

IVIM parameters	WM	CGM	DGM
*f* [%]	4.21 ± 1.27 (4.19–4.23)	5.04 ± 2.12 (5.02–5.06)	5.22 ± 1.59 (5.17–5.26)
*D* [μm^2^/ms]	0.818 ± 0.127 (0.816–0.820)	1.042 ± 0.358 (1.038–1.046)	0.819 ± 0.202 (0.813–0.830)
*ν* [mm/s]	1.30 ± 0.77 (1.29–1.31)	1.63 ± 0.75 (1.62–1.64)	1.20 ± 0.68 (1.17–1.23)
*τ* [ms]	130 ± 49 (130–131)	117 ± 49 (117–118)	136 ± 47 (135–138)

Abbreviations: *ν*, blood flow velocity; *τ*, correlation time; *D*, diffusion coefficient; *f*, perfusion fraction.

## 5 Discussion

In this study, we present an analytical IVIM model applicable across temporal blood flow regimes and for diffusion encoding gradient waveforms enabling flow compensation. The incorporation of flow compensation into the model provides improved sensitivity to the encoding time dependence relative to the model proposed by Kennan et al., while the analytical expression gives improved interpretability relative to the simulation‐based model suggested by Wetscherek et al. [2, 4]. Experiments utilizing flow‐compensated and non‐flow‐compensated diffusion encoding, similar to those of Ahlgren et al. and Jalnefjord et al. [6, 23], were extended to also cover a range of encoding times such that the correlation time and velocity of capillary blood flow could be estimated in the healthy human brain. Results derived with the proposed model are directly transferable to established models, as the proposed model approaches these in the temporal limits of very long or short encoding times.

### 5.1 Proposed Model

Two main velocity autocorrelation functions (VACF), linear and exponential, have been explicitly or implicitly suggested for IVIM modeling. The conventional model assumes straight capillary segments of equal length, corresponding to a linear VACF [1]. This is also the underlying model assumed by Wetscherek et al. in their simulation‐based framework [4]. The use of a linear VACF is, however, not generally compatible with the Gaussian phase approximation used, for example, in the current work [4]. Instead, an exponential VACF was selected here, for which the blood is under continuous directional change. This results in a phase distribution compatible with the Gaussian phase approximation, facilitating the derivation of an analytical model. Attempting to identify a definite VACF for a given tissue is challenged by the inherent noise in the signal of an IVIM experiment, likely rendering such precision redundant. Similarly, the true distribution of velocities, here assumed to be Gaussian but which may be of other types due to variable vessel size and pipe flow distributions, is in practice as difficult to determine. Nevertheless, the availability of an analytical model can increase the general understanding of how the MR signal is affected by different pulse sequence parameters for different tissue types (sets of IVIM parameters). This can, in turn, enable the use of a broader range of optimization methods, including those requiring an analytical forward model, to generate more time‐efficient imaging protocols [24, 25, 26].

Estimating the IVIM correlation time requires a pulse sequence that allows for adjusting the timing of the diffusion encoding, preferably with a clearly defined diffusion encoding time parameter. We here derived a model for an FC/NC DDE to exploit the signal dynamics obtained in FC compared to NC in a joint analysis, similar to Ahlgren et al. [6], but for multiple encoding times. Such a pulse sequence enables changing the encoding time while keeping all other pulse sequence parameters fixed (i.e., *b*‐value, *c*‐value, δ and Δ). The algorithm used here to derive the model (described in the Appendix A) is, however, applicable to any pulse sequence used for diffusion encoding as long as *G*(*t*) can be described by piecewise analytical functions. Future work using other pulse sequences should thus benefit from the current work, which provides a simplified path towards a specific signal model. Alternative pulse sequences include PGSE for NC gradients or oscillating gradient spin echo (OGSE). PGSE can allow for shorter encoding times than DDE; however, NC‐PGSE and the corresponding gradients for FC experiments, as used by Wetscherek et al. [4], do not share common waveforms, which complicates a model for joint analysis. OGSE employs periodic gradients, effectively reducing the diffusion time while mitigating eddy currents [27, 28]. The encoding time is not well‐defined for OGSE but can instead be expressed in terms of the oscillating frequency [5, 29].

The FC signal reveals a non‐monotonic behavior with respect to *τ* (Figure 3c). A minimum can be seen for correlation times just short enough for the FC gradients not to result in a partial rephasing of the signal (*T*/τ ˜3–6). For longer τ, partial or full rephasing, and thus an increased signal, is achieved when transitioning towards the ballistic regime. On the other hand, for shorter τ, the FC signal increases for decreasing τ, following the decreasing effective D* in this regime. As a result, two distinct tissues with long and short τ, respectively, may yield similar FC signals. This ambiguity may be resolved by a contrasting NC signal and sampling at multiple *T* (where the latter is required to separate τ and $\left\langle {\bar{\nu }}^{2}\right\rangle$). This non‐monotonic behavior is presented to give theoretical insight into the model and is not to be confused with the signal decay seen when acquiring signal for multiple *T* (Figure 3b). In the practical case, where the properties of the flow are fixed and multiple *T* are sampled, increasing *T*/τ allows the flow to change direction an increasing number of times. This results in a smaller net displacement, and consequently, the NC signal experiences less signal attenuation. In contrast, the FC signal partially rephases the signal until it reaches the pseudodiffusive limit at *T*/τ ≥4, at which it converges with the NC signal.

Analytical models are useful as they provide explicit relationships between variables. They enable a general interpretation of the governing contrast mechanisms, provided the underlying assumptions hold. The IVIM pseudodiffusive model is attractive due to its simplicity and straightforward implementation. However, it requires a sufficiently long encoding time to avoid misinterpretation. The previously reported number of direction changes required to establish a pseudodiffusive regime varies between 4 and 10 [1, 2, 4, 6], likely in part due to differences in experimental setup (e.g., definition of the encoding time parameter, blood velocity). The simulations conducted in this study produce similar results, suggesting that the pseudodiffusive regime requires *T*/τ ≥ 4 (Figure 3b). Similarly, a separation between the FC and NC signal could be observed in the in vivo data, indicating that a diffusive regime was not established (Figure 4). Aiming for a ballistic model places a higher demand on the scanner's performance as shorter encoding times are needed. Wu et al. demonstrated an encoding time dependence of the IVIM signal in the mouse brain at very low diffusion times using OGSE [5], suggesting that a ballistic regime was not established.

### 5.2 Estimated IVIM Parameter Values

Simulation results indicate that parameter estimation based on the proposed model is SNR‐demanding, especially with regard to estimating the correlation time τ (Figure 5). This presents a particular challenge in the healthy brain, where the perfusion signal is low. The simulations based on IVIM parameter values typical for the brain show that the bias for *D*, *f*, and *v* is small enough for a relatively straightforward interpretation, whereas the estimated values of *τ* must be interpreted with caution.

A positive estimation bias for τ was seen for the diffusive and intermediate regimes, while a negative bias was seen in the ballistic case. These results point towards a true *τ* of < 100 ms for an estimated *τ* = 130 ms (Table 1). This would be in line with the cerebral capillaries of anesthetized cats, where the median velocity of red blood cells was 1.5 mm/s with a median segment length of 108 μm, corresponding to a τ around 70 ms [30]. It would be somewhat shorter than the τ in human liver and pancreas, which Wetscherek et al. reported to be 144 ± 10 and 224 ± 4 ms, respectively [4], although this potential difference should be interpreted with caution, given the high uncertainty and potential bias inherent to the τ estimated for the brain. It is, however, worth noting that this bias in estimated τ is not inherent to the proposed model but rather a result of the challenging conditions in the healthy brain with a very low perfusion signal, as shown by the simulations of more highly perfused organs, such as the liver and pancreas, in the supporting material (Figure S1). While the simulation results presented in Figure 5 show the difficulty in distinguishing between different τ < 100 ms at low to moderate SNR levels, in vivo data shown here and previously by Ahlgren et al. [6] and Jalnefjord et al. [23] display a signal separation between the FC and NC signal, indicating that the diffusive regime is not valid. Altogether, this suggests that the incoherent flow probed at encoding times 50–100 ms, as in the current study, pertains to an intermediate flow regime, although the precise value of τ in the healthy human brain remains to be determined.

The blood flow velocity was estimated here to be around 1–2 mm/s. In similar studies, utilizing an FC/NC DDE sequence and applying a ballistic model, Ahlgren et al. [6] and Jalnefjord et al. [23], estimated a cerebral velocity dispersion $\nu_{d}$ to around 1.7 and 1.5 mm/s, respectively, which relates to ν as $\nu_{d}^{2}=\frac{\left\langle {\bar{\nu }}^{2}\right\rangle }{2d}$. This corresponds to higher values of ν than estimated in this study, and may be explained by a bias towards higher values due to parameter regularization during model fitting [6], the influence of a bimodal distribution [23], and by the observed negative bias of ν at realistic noise levels in this study (Figure 5). Capillary blood velocity in the liver has been reported to be around 1.7 mm/s [31], and > 3 mm/s through arterioles/venules [4, 31].

The estimated values of *f* were in this study around 4%–5% (Table 1). This is higher than previous studies using joint analysis of FC/NC DDE data with a ballistic model, where *f* instead was reported to be around 1.5%–2.5% [6, 23]. These results may indicate that for encoding times achievable on a clinical MR system (37.7 and 50 ms for a maximum *b*‐value of 200 s/mm^2^ for Ahlgren et al. and Jalnefjord et al., respectively), the assumption of a ballistic capillary blood flow is not completely fulfilled. The result would be a smaller signal difference between FC and NC data (Figure 3b) and, accordingly, an underestimation of *f* if a model assuming the ballistic regime is used. On the other hand, in studies utilizing conventional diffusion encoding and IVIM analysis, reported values of *f* were typically around 7%–11% [32]. The discrepancy between those results and the ones presented here may be due to the choice of higher *b*‐values. The influence of non‐Gaussian diffusion can be substantially more pronounced at *b*‐values around 1000 s/mm^2^ (typically used for conventional diffusion encoding) than at 200 s/mm^2^, which was used in the current study [33]. If non‐Gaussian diffusion is not accounted for when using higher *b*‐values, this may result in an overestimation of *f* [6].

### 5.3 Limitations

This study has several limitations to consider. First, due to the limited number of participants, our results should be regarded as preliminary and require validation in a larger cohort. Second, the diffusion coefficient of blood was fixed (1.75 mm^2^/s) in this study to improve the robustness of parameter estimation [6], however, it is known to vary with hematocrit level and the encoding scheme can influence its estimate [34]. Finally, acquiring NC and FC DDE with multiple encoding times is time‐consuming, and a potential clinical application requires scan time optimization. Future work should focus on improving the efficiency of the acquisition to reduce scan duration and potentially mitigate estimation bias by a more effective utilization of the available SNR, e.g., through Cramér‐Rao lower bound optimization and tensor denoising [25, 35].

## 6 Conclusion

Based on a stochastic differential equation, an analytical model could be derived for the intermediate IVIM regime, which can be simplified to limit cases commonly seen in the literature (ballistic and diffusive regimes, respectively). Simulations and in vivo estimates of microvascular correlation times and velocities suggest that IVIM is in the intermediate to ballistic regime in the healthy human brain for encoding times achievable on clinical MRI systems, contrary to what is commonly assumed.

## Funding

This work was supported by Sahlgrenska University Hospitals Research Foundations, Cancerfonden, 211568 Pj, 243750 Pj, Stiftelsen Assar Gabrielssons Fond, Swedish Governmental Funding of Clinical Research (ALF), Stiftelsen Jubileumsklinikens Forskningsfond mot Cancer, 2022:424, Åke Wiberg's Foundation, Kungl. Vetenskaps‐ och Vitterhets‐Samhället i Göteborg.

## Conflicts of Interest

The authors declare no conflicts of statement.

## Supporting information


**Figure S1.** Estimated IVIM parameters for synthetic data at different SNR levels, with signal generated using realistic IVIM parameters for (a) brain, (b) liver [4], and (c) pancreas [4]. The horizontal black dashed line shows the ground‐truth value, the vertical black line shows the interquartile range, and the orange line shows the median. Simulations for well‐perfused liver and pancreas show substantially less noise sensitivity compared to simulations for brain, which has an intrisically low perfusion signal.

## Data Availability

Python code for IVIM parameter estimation using the proposed model is available at https://github.com/oscarjalnefjord/ivim/tree/intermediate_regime (commit bc6a0b8).

## Appendix A

Equation (8) was derived using sympy 1.12 for symbolic math operations. While the gradient waveforms G(t) and the resulting dephasing q(t) can be defined as piecewise functions in sympy, direct integration of Equation (7) proved practically infeasible. Instead, the integration was split into the time intervals for which G(t) could be defined as a simple continuous function, and the resulting integral was sequentially added. Absolute value operations obtained from the exponential VACF were converted into if clauses. The algorithm is schematically shown here:

1. Initialize $\left\langle \phi^{2}\right\rangle =0$
2. Loop over time steps i=0,1,2,…, between which *G*(*t*) can be defined as a continuous function.
  - Define $q_{i}\left(t\right)$, $t_{i}$ and $t_{i+1}$ for the time interval $t\in \left[t_{i},t_{i+1}\;\right]$.
  - Loop over time steps j=0,1,2,…, between which $G\left(t^{\prime }\right)$ can be defined as a continuous function
    - Define $q_{j}\left(t^{\prime }\right),t_{j}^{\prime }$ and $t_{j+1}^{\prime }$ for time interval $t_{j}^{\prime }\in \left[t_{j}^{\prime },t_{j+1}^{\prime }\right]$. This step repeats step (a) but for the outer variable of integration
    - Compute the partial contribution of the mean squared phase, ${\left\langle \phi^{2}\right\rangle }_{i,j}$
      • If i>j
        $${\left\langle \phi^{2}\right\rangle }_{i,j}=\int_{t_{j}^{\prime }}^{t_{j+1}^{\prime }}\int_{t_{i}}^{t_{i+1}}q_{j}\left(t^{\prime }\right)q_{i}\left(t\right)e^{-\frac{t-t^{\prime }}{\tau }}dtdt^{\prime }$$
      • If i<j
        $${\left\langle \phi^{2}\right\rangle }_{i,j}=\int_{t_{j}^{\prime }}^{t_{j+1}^{\prime }}\int_{t_{i}}^{t_{i+1}}q_{j}\left(t^{\prime }\right)q_{i}\left(t\right)e^{-\frac{t^{\prime }-t}{\tau }}dtdt^{\prime }$$
      • If i=j, partition the integral domain into two parts
        $$\begin{array}{ll} I_{i,j}^{\left(1\right)} & =\int_{t_{j}^{\prime }}^{t_{j+1}^{\prime }}\int_{t_{i}}^{t^{\prime }}q_{j}\left(t^{\prime }\right)q_{i}\left(t\right)e^{-\frac{t^{\prime }-t}{\tau }}dtdt^{\prime }\;t\leq t^{\prime }\\ I_{i,j}^{\left(2\right)} & =\int_{t_{j}^{\prime }}^{t_{j+1}^{\prime }}\int_{t^{\prime }}^{t_{i+1}}q_{j}\left(t^{\prime }\right)q_{i}\left(t\right)e^{-\frac{t-t^{\prime }}{\tau }}dtdt^{\prime }\;t>t^{\prime }\\ {\left\langle \phi^{2}\right\rangle }_{i,j} & =I_{i,j}^{\left(1\right)}+I_{i,j}^{\left(2\right)} \end{array}$$

iii. Update $\left\langle \phi^{2}\right\rangle =\left\langle \phi^{2}\right\rangle +{\left\langle \phi^{2}\right\rangle }_{i,j}$
