# Supplementary material for: Modeling the Intermediate Flow Regime in Flow‐Compensated Intravoxel Incoherent Motion MRI
Source: Magn Reson Med. 2026 Jan 26;95(6):3476–87. doi: 10.1002/mrm.70267 (PMC13049275; doi:10.1002/mrm.70267)
Supplement: Supplementary file 1 — Figure S1. Estimated IVIM parameters for synthetic data at different SNR levels, with signal generated using realistic IVIM parameters for (a) brain, (b) liver [4], and (c) pancreas [4]. The horizontal black dashed line shows the ground‐truth value, the vertical black line shows the interquartile range, and the orange line shows the median. Simulations for well‐perfused liver and pancreas show substantially less noise sensitivity compared to simulations for brain, which has an intrisically low perfusion signal. [file MRM-95-3476-s001.docx]

Supporting Information


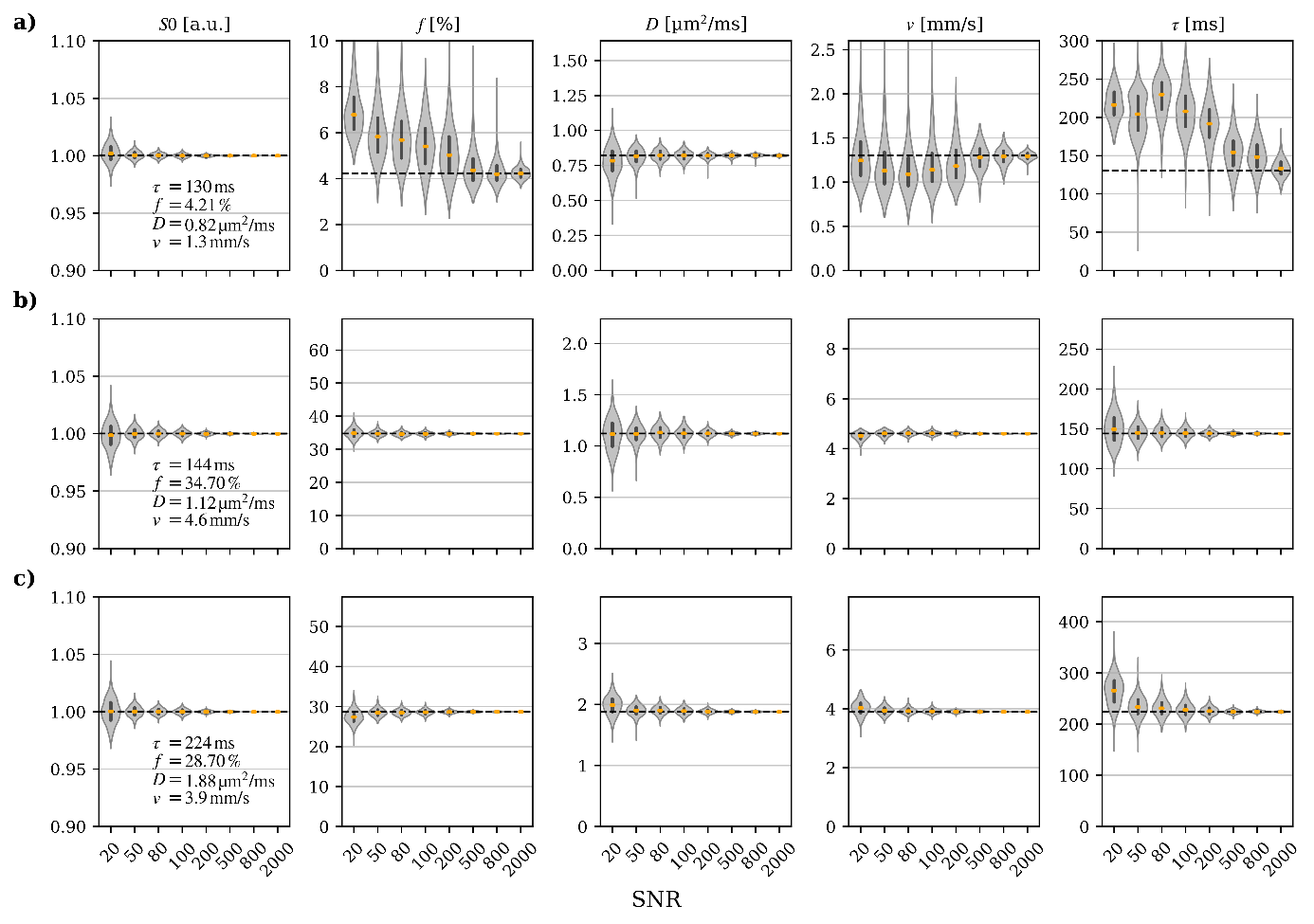


**Supporting Information Figure S1**: Estimated IVIM parameters for synthetic data at different SNR levels, with signal generated using realistic IVIM parameters for **a)** brain, **b)** liver ^4^, and **c)** pancreas ^4^. The horizontal black dashed line shows the ground-truth value, the vertical black line shows the interquartile range, and the orange line shows the median. Simulations for well-perfused liver and pancreas show substantially less noise sensitivity compared to simulations for brain, which has an intrisically low perfusion signal.
